# The Utilization, Application, and Impact of Institutional Special Needs Plans (I-SNPs) in Nursing Facilities: A Rapid Review

**DOI:** 10.3390/healthcare14010071

**Published:** 2025-12-27

**Authors:** Michael Mileski, Roland Shapley, Bradley Beauvais, Joseph Baar Topinka, Ramalingam Shanmugam, Jose A. Betancourt, Matthew Brooks, Rebecca McClay

**Affiliations:** 1 School of Health Administration, Texas State University, San Marcos, TX 78666, USA; rshapley@txstate.edu (R.S.); bmb230@txstate.edu (B.B.); josephtopinka@txstate.edu (J.B.T.); shanmugam@txstate.edu (R.S.); jose.a.betancourt@txstate.edu (J.A.B.); mbrooks@txstate.edu (M.B.); 2 School of Science, Technology, Engineering, and Math, American Public University System, Charles Town, WV 25414, USA; rebecca.mcclay@gmail.com

**Keywords:** institutional special needs plans (I-SNPs), Medicare Advantage, nursing facilities

## Abstract

**Background/Objectives**: Institutional Special Needs Plans (I-SNPs) are designed to enhance the quality of care for long-term nursing facility (NF) residents. However, utilization patterns vary significantly, and their broader impact remains only partially understood. This rapid review aims to identify, map, and synthesize the existing literature on the use of I-SNPs in nursing homes. **Methods**: Following Arksey and O’Malley’s framework and PRISMA-ScR guidelines, we conducted a comprehensive search of academic and gray literature using a predefined Boolean string. The extracted data were organized and analyzed thematically. **Results**: The synthesized literature (n = 12 studies) revealed four primary themes: (1) Market Penetration and Enrollment; (2) Models of Care Application; (3) Impact on Clinical and Financial Outcomes; and (4) Barriers to Utilization. **Conclusions**: I-SNP utilization represents a shift from fragmented FFS payment models toward integrated managed care within nursing facilities. Evidence shows a reduction in acute care transfers, although findings for other outcomes are mixed, underscoring the need for further research and policy development.

## 1. Introduction

### 1.1. Rationale

The quality of care for long-term residents in nursing facilities (NFs) remains a critical national challenge, an issue deemed a “national imperative” [1]. This population, characterized by advanced age, multiple chronic conditions, and significant functional and cognitive impairment, frequently experiences fragmented care, poor health outcomes, and high rates of potentially avoidable hospitalizations [2]. These acute care transfers are not only costly but are known to be disorienting for residents, often precipitating functional decline [3].

For decades, the traditional Fee-for-Service (FFS) Medicare system has been identified as a key driver of this fragmentation. FFS often produces misaligned financial incentives, rewarding high-intensity acute care (e.g., hospitalization) rather than investment in proactive, on-site preventive or primary care services [3]. Nursing facilities operating under this model have minimal financial incentive to invest in enhanced on-site clinical models, as the facilities themselves would bear the cost of these services while the financial savings from reduced hospitalizations would accrue entirely to the Medicare program [4].

In this context, Institutional Special Needs Plans (I-SNPs) were established by the Medicare Modernization Act of 2003. I-SNPs are a specialized, capitated type of Medicare Advantage (MA) plan exclusively enrolling beneficiaries who require an institutional level of care and reside, or are expected to reside, in a long-term care facility for 90 days or more [5]. These plans are designed to address the shortcomings of FFS by integrating Medicare (and often Medicaid) financing and providing a specific Model of Care (MOC). This MOC is intended to be a high-touch, coordinated, and clinician-led application of resources directly within the nursing facility [6,7]. Under this model, the I-SNP entity assumes financial responsibility for the resident’s comprehensive medical care (and potentially their long-term care if they are dually eligible for Medicaid) [3,8]. This financial integration provides the plan with a direct incentive to invest in on-site primary and acute care, thereby preventing costly, avoidable hospitalizations.

Despite their theoretical advantages and two decades of existence, I-SNP penetration remains relatively low, covering approximately 12% of eligible Medicare beneficiaries in nursing homes [3]. Furthermore, the utilization of these plans—referring not just to enrollment numbers but to the fidelity and application of their care models and their ultimate use in improving resident care—is not well understood. Substantial variation exists in how these plans are implemented and in the outcomes they produce. Therefore, a comprehensive mapping of the existing literature is required.

This rapid review addresses the question: What is the extent, nature, and character of the existing literature on the utilization, application, and use of I-SNPs within nursing facilities?

### 1.2. Objectives

The purpose of this rapid review is to identify how I-SNPs are utilized in practice, evaluate approaches to implementation, and highlight potential drawbacks associated with their use. This paper specifically examines the utilization of I-SNPs, and it is hoped that this study will provide evidence-based guidance for the rapid implementation of these insurance plans in nursing homes.

### 1.3. Population, Concept, and Context (PCC)

As this paper examines a broad landscape of information regarding iSNP implementation, researchers utilized the PCC concept to better define our inclusion criteria.

Population: The population for this study consists of those nursing homes that either utilize or may potentially utilize I-SNP plans for their Medicare utilization under an Advantage plan. This extends to the entire patient population of Medicare Advantage recipients who utilize a plan that offers the ability to enroll in I-SNP benefits.

Concept: This rapid review focuses specifically on the benefits and drawbacks for nursing home residents utilizing I-SNP plans.

Context: This rapid review primarily focuses on nursing homes; however, a small number of other healthcare environments are referenced when relevant due to the limited amount of published research specific to I-SNP utilization.

## 2. Materials and Methods

### 2.1. Overview

The research process began with a review of the manuscript’s intent by the researchers involved, as well as a review of pertinent healthcare terminology. To guide the review, we applied PRISMA-ScR standards [9], the Kruse protocol [10], and Arksey and O’Malley’s framework for scoping studies [11]. Five databases were queried to complete the literature review, including PubMed, CINAHL Ultimate, Google Scholar, Academic Search Complete, and Nursing and Allied Health Reference Source. A simplified three-term Boolean search was chosen to maximize yield given the limited depth of existing literature on I-SNPs.

### 2.2. Inclusion Criteria

Study search terms and Boolean operators generated the database search string that resulted in the selection of articles for inclusion in the manuscript. The Boolean string utilized was (“institutional special needs plan” OR “i-SNP” OR “iSNP”) AND (“utilization” OR “application” OR “use” OR “usage”) AND (“nursing home” OR “nursing facility” OR “SNF” OR “NF”). This broader terminology was adopted after initial testing showed that narrower strings excluded relevant articles. The initial search yielded 516 articles published between 1 January 2003, and 20 December 2025. This date range was used to cover the time since the inception of the iSNP. For the purposes of our research, gray literature was defined as any material collected outside of a peer-reviewed journal. This includes information from insurance companies and Congressional advisories.

### 2.3. Exclusion Criteria

Studies were included in the review if they mentioned any terms surrounding the Boolean strings and were healthcare-related. Due to the limited number of peer-reviewed publications, relevant gray literature was included when it made a substantive contribution to the topic.

**Figure 1:**
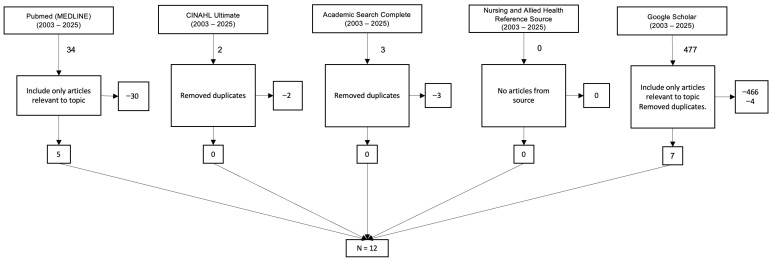
Diagram that demonstrates the study selection process.

Additional filters were applied to refine results. Articles were included only when all researchers agreed on their relevance. PubMed yielded five articles after exclusions. CINAHL Ultimate and Academic Search Complete each returned no unique articles after duplicates were removed. The Nursing and Allied Health Reference Source yielded no articles. Google Scholar contributed seven articles after removing duplicates, resulting in a total of twelve final studies. Figure 1 shows the diagram outlining the article selection process.

The authors conducted a rigorous review of the twelve articles chosen for inclusion in their analysis by reading the full manuscripts of each article. At least three researchers agreed that any information extracted from individual articles would be included in the analysis. No discrepancies were reported among the researchers during the review of articles for inclusion or exclusion. Because the pool of eligible studies was limited, a formal assessment of bias was not conducted; however, the research team identified no overt indicators of bias.

## 3. Results

### 3.1. Overview

Institutional Special Needs Plans (I-SNPs) are a specialized type of Medicare Advantage (MA) plan specifically designed for individuals who reside in or are expected to need the level of services provided in a long-term care facility for 90 days or longer. The model aims to improve care quality and reduce avoidable expenses, primarily by using capitated payments and employing on-site advanced practice clinicians (such as nurse practitioners) for primary care, care planning, and care coordination.

The evaluation of I-SNPs in nursing homes reveals several substantial benefits, particularly concerning acute care utilization, alongside specific drawbacks related to functional status decline and quality concerns in other domains.

Table 1 presents the coding of the studies, along with their strength and quality, according to the Johns Hopkins Nursing Evidence-Based Practice Model (JHNEBP). While the study design is generally an essential tenet of inclusion in this type of manuscript, it was not considered here due to the small number of available studies for consideration.

The quality results of the identified studies, as assessed by the JHNEBP methodology, demonstrate that the majority of the articles (58.3%) came from the level II (quasi-experimental studies) category. There was one level III category (8.3%, non-experimental or qualitative studies) article and four level IV category (33.3%, opinion based on clinical evidence) articles used in the study. Given the limited volume of available literature, the research team included all relevant materials regardless of study design, while acknowledging variability in methodological rigor.

### 3.2. Thematic Findings

The analysis of the included literature revealed four primary themes regarding the utilization of I-SNPs in nursing facilities.

#### 3.2.1. Theme 1: Market Penetration and Enrollment (The Extent of Use)

This theme describes the landscape of I-SNP availability and enrollment. The literature indicates that I-SNPs remain a relatively small (approx. 177 plans in 2024) but consistently growing segment of the MA market [7]. Enrollment has shown steady growth (approximately 7% year-over-year from 2021 to 2024), despite the absolute number of plans experiencing minor decreases [6,7]. This growth is notably more rapid in rural than in urban areas, though the majority of enrollment remains urban [7,14]. I-SNP disenrollment rates were found to be low, potentially indicating a higher degree of plan satisfaction among members [17].

The literature also distinguishes between “Institutional-only” plans and “Institutional Equivalent” (IE-SNPs), with the latter serving beneficiaries who live in the community (e.g., assisted living) but require an institutional level of care [4,7]. This distinction is critical for understanding market data, as enrollment growth has been driven by both categories [7].

Nursing homes with I-SNP residents tend to be larger on average (134.5 beds versus 95.5 beds), more likely to be for-profit (76.6% versus 68.5%), and more likely to be part of a chain [18]. These facilities also had a slightly lower average overall quality rating (3.0 stars versus 3.2 stars) and lower total staffing levels (3.6 total staffing hours per resident day versus 4.0 h) as compared to those without I-SNP enrollees [18]. Enrollment in I-SNPs is highly concentrated among medically complex and high-need populations, particularly in dual-eligibles (93.8%) [18]. Overall, the literature suggests that while I-SNP enrollment is expanding, growth remains uneven across geographic regions and facility types.

#### 3.2.2. Theme 2: The I-SNP Model of Care (The Application of Use)

This theme details how I-SNPs are operationalized. The literature is clear that I-SNP “utilization” is synonymous with the application of a specific Model of Care (MOC) [6,13]. This MOC is the core mechanism of the plan, designed to manage high-need residents.

Key components of this MOC application include:

- On-site Clinicians: The model’s foundation is the use of dedicated advanced practice clinicians, primarily Nurse Practitioners (NPs) and Physician Assistants (PAs), who are embedded within the nursing facility [4,8,13,14,16,18]. Some models refer to these clinicians as “NF-ists” (Nursing Facility-ists) [13].
- Care Coordination: These on-site clinicians provide enhanced primary, acute, and preventive care [13]. They conduct comprehensive Health Risk Assessments (HRAs) and develop Individualized Care Plans (ICPs) for each member [6,13,14,16].
- Proactive Management: The goal is proactive care management to identify and treat new conditions or symptoms (e.g., pain, depression, infection) early, thereby preventing clinical deterioration [4,6]. This involves improved communication among the plan, the facility staff, the resident, and their family [6]. Together, these elements reflect a high-touch clinical model distinct from traditional physician-driven care in nursing homes.

#### 3.2.3. Theme 3: Impact of Utilization on Clinical and Financial Outcomes (The Effect of Use)

This was a robustly detailed theme in the literature, focusing on the results of the I-SNP application.

- Reduced Hospitalizations: A consistent finding across multiple studies is that I-SNP utilization is associated with significant reductions in avoidable hospitalizations and emergency department (ED) visits [4,6,12,14,16,18]. One major study found that I-SNP members had 38% fewer hospitalizations and 51% lower ED use compared to FFS beneficiaries [4,14]. Another analysis found that facilities with mature I-SNP penetration (high enrollment) experienced a 4.1 percentage point decline in hospitalizations [12,14,16,18].
- Mechanism of “Substitution”: The literature clearly identifies the mechanism for this reduction: substitution. I-SNP models use on-site skilled nursing care in place of hospital care [13]. This is enabled by a crucial policy feature: the waiver of the 3-day qualifying hospital stay requirement [4,14,16,18]. This allows NPs/PAs to provide and bill for skilled services within the facility. This explains the finding that I-SNP members had 112% higher SNF utilization—delivered within their existing facility rather than after hospitalization [4].
- Mixed Clinical Outcomes: While the impact on hospitalizations is clear, the evidence for other clinical outcomes is nuanced and complex. One large-scale study found that I-SNP maturity was associated with positive outcomes (fewer pressure ulcers, fewer urinary tract infections) [14,16]. However, the same study found associations with negative outcomes: increased need for help with Activities of Daily Living (ADLs), increased use of antipsychotics, and more falls [12]. For residents with Alzheimer’s disease and related dementias (ADRD), reduced acute care use could generate an estimated $1.2 billion in annual savings through lower hospitalization rates [18].
- End-of-Life Care: I-SNP utilization also impacts other care processes. One national analysis found that I-SNP availability was associated with changes in hospice utilization patterns, though this relationship varied by facility size [12,15].

#### 3.2.4. Theme 4: Barriers to Utilization (The Limits of Use)

Finally, the literature identifies significant barriers that hinder the broader adoption of I-SNPs.

- Financial Misalignment: A primary barrier is the competing financial incentive in traditional FFS Medicare, which can make hospitalization more profitable for some NFs than investing in the on-site care models I-SNPs require [3,4].
- Payer and Provider Reluctance: Utilization is limited because some MA organizations (payers) may not want to contract with all NFs, and some NFs (providers) may not wish to participate in an I-SNP network [3]. Nearly 70 percent of nursing homes did not have any residents enrolled in I-SNPs [14]. I-SNPs were also not available for enrollment in more than 60 percent of counties in the United States [14].
- Workforce: (Inferred) The successful application of the MOC is dependent on a specialized workforce of NPs and PAs trained in geriatrics, which may be a limiting factor in some markets [4,6].
- Negative clinical outcomes: An increase of 5.3 pp in the need for help with activities of daily living may suggest a decline in residents’ functional ability and increased dependence for those enrolled in these plans [16]. There are also small increases in the use of antipsychotic medications (1.3 pp), falls (1.0 pp), and the use of physical restraints (1.0 pp) [16]. I-SNP enrollment was also associated with greater use of lower-rated hospice providers [15].

## 4. Discussion

This rapid review mapped a robust body of literature detailing the utilization of I-SNPs in nursing facilities. The central finding is that I-SNP “utilization” reflects not merely enrollment but the active deployment of a clinician-led, on-site MOC intended to substitute for hospital-based acute care [4,6,13].

### 4.1. The I-SNP Substitution Model

The evidence strongly supports the primary value proposition of I-SNPs: reducing avoidable hospitalizations [3,4,12]. The combination of on-site advanced practice clinicians and the 3-day stay waiver forms a powerful clinical and policy mechanism for substituting facility-based skilled care for hospital care. The associated rise in SNF utilization is therefore a feature—not a flaw—of the model [4].

### 4.2. The “Mixed Outcomes” Conundrum

A critical finding from this review is the mixed data on other clinical outcomes [12]. The reported association between I-SNP maturity and negative outcomes (falls, ADL decline, antipsychotic use) is alarming and demands interpretation [12]. This association does not necessarily prove I-SNPs provide worse care in these domains. Several alternative hypotheses must be considered:

1. Adverse Selection: I-SNP plans may attract or retain residents with more complex needs and higher functional decline than the FFS comparison group [12,13].
2. Surveillance and Documentation Bias: The presence of on-site, dedicated NPs [4,13] likely results in superior identification and documentation of these conditions (falls, ADL changes) compared to FFS residents, who may only be seen by a physician intermittently. This would make the I-SNP group appear to have worse outcomes, when in fact their care is simply more closely monitored.
3. Model Deficiencies: It is possible that the I-SNP MOC, while excelling at acute care management, is less focused on restorative, functional, or dementia care, leading to genuine declines in these areas [4,6,12,13].

Collectively, these findings suggest the need for refinements to ensure whole-person care alongside acute care management.

### 4.3. Gaps in the Literature

This review identified several significant gaps. There is a distinct lack of research on:

- Resident and family satisfaction with I-SNP models.
- The effectiveness of different MOCs (e.g., staffing ratios, NP vs. PA, “NF-ist” model).
- Longitudinal studies that can untangle the “mixed outcomes” finding (e.g., to test the surveillance bias hypothesis).
- Qualitative research exploring the facility-level barriers to adoption [3].

## 5. Conclusions

This rapid review highlights that Institutional Special Needs Plans (I-SNPs) represent a critical evolution in the financing and delivery of long-term care, shifting the paradigm from fragmented Fee-for-Service models toward integrated, value-based care. The synthesis of the available literature confirms that the I-SNP model successfully achieves its primary strategic objective: the reduction in avoidable hospitalizations and emergency department visits through the “substitution” of acute hospital care with skilled, on-site clinical management. By deploying advanced practice clinicians directly into nursing facilities and utilizing policy mechanisms like the 3-day stay waiver, I-SNPs effectively treat residents in place, offering a viable solution to the costly disruptions associated with acute care transfers.

However, the utilization of I-SNPs is not without complexity. While the model excels at acute care management, evidence regarding broader clinical outcomes remains mixed, with some data suggesting associations with functional decline and increased antipsychotic use. Whether these findings stem from adverse selection, surveillance bias due to increased clinician presence, or genuine gaps in the model’s focus on restorative care requires urgent investigation. Furthermore, barriers such as financial misalignment, workforce shortages, and varying market penetration suggest that the benefits of I-SNPs are not yet equitably accessible across all geographic regions or facility types.

Ultimately, I-SNPs offer a promising framework for improving the coordination of care for high-need nursing home residents. To maximize their potential, future policy and practice must ensure that the incentivization of reduced hospitalizations does not inadvertently overshadow the need for holistic, maintenance-based nursing care. Future research must prioritize longitudinal studies and qualitative inquiries into resident satisfaction to fully understand the trade-offs involved in this managed care model.

### Implications for Policy and Practice

The findings of this review suggest several actionable pathways for policymakers and nursing facility administrators. First, the misalignment of financial incentives remains a primary barrier to broader adoption; traditional Fee-for-Service (FFS) models often reward high-intensity acute care rather than the preventive, on-site management prioritized by I-SNPs. Policy efforts should focus on harmonizing these incentives to encourage facilities to invest in the necessary on-site clinical infrastructure.

Second, the success of the I-SNP model is intrinsically linked to the availability of a specialized workforce, specifically Nurse Practitioners and Physician Assistants trained in geriatrics. As the demand for these plans grows, there is a concurrent need for educational pipelines and workforce development strategies to ensure a sufficient supply of clinicians capable of executing this high-touch Model of Care.

Finally, the “mixed outcomes” identified in this review—specifically the contrast between reduced hospitalizations and potential declines in functional status—indicate that current quality metrics may need refinement. Regulatory bodies and plan administrators should consider expanding quality measures to ensure that the aggressive management of acute conditions does not come at the expense of restorative care, ADL maintenance, or the reduction in antipsychotic utilization.

## 6. Study Limitations

This study is limited by the small volume of timely research available on I-SNPs. Much of the included literature scored well on the JHNEBP scale; however, the review also incorporated gray literature, some of which lacked methodological detail, which may limit interpretability. This paper did not conduct a formal risk-of-bias assessment due to the already limited number of available articles. This reliance on gray literature could have introduced a level of bias into the work. Further research with stronger study designs is needed to more definitively assess the benefits and drawbacks of I-SNP utilization.

## Figures and Tables

**Table 1:** Johns Hopkins Nursing Evidence-Based Practice Model ratings for each article.

Year	Author	Strength	Quality
2019	Dhingra et al. [12]	II	A
2019	McGarry and Grabowski [4]	II	A
2020	Great Plains Medicare Advantage [6]	IV	B
2021	Singletary et al. [8]	II	A
2021	Signature Advantage [13]	IV	B
2024	Chen et al. [14]	III	A
2024	Yeh and Yen [7]	II	A
2024	White et al. [15]	II	A
2025	Chen and Grabowski [16]	II	A
2025	Yun et al. [17]	II	A
2025	Rahman et al. [18]	IV	B
2025	MedPAC [3]	IV	B

Shaded authors are gray literature which was included.

## Data Availability

No new data were created or analyzed in this study. Data sharing is not applicable to this article.

## References

[1] National Academies of Sciences, Engineering, and Medicine, Health and Medicine Division, Board on Health Care Services, Committee on the Quality of Care in Nursing Homes (2022). The National Imperative to Improve Nursing Home Quality: Honoring Our Commitment to Residents, Families, and Staff.

[2] Grabowski D.C., Chen A., Saliba D. (2023). Paying for Nursing Home Quality: An Elusive But Important Goal. Public. Policy Aging Rep..

[3] MedPAC (2025). Chapter 5: Medicare Beneficiaries in Nursing Homes (June 2025 Report).

[4] McGarry B.E., Grabowski D.C. (2019). Managed Care for Long-Stay Nursing Home Residents: An Evaluation of Institutional Special Needs Plans. Am. J. Manag. Care.

[5] US Centers for Medicare and Medicaid Services Special Needs Plans (SNP)|Medicare. https://www.cms.gov/medicare/enrollment-renewal/special-needs-plans.

[6] (2020). Great Plains Medicare Advantage. Model of Care.

[7] Yeh M., Yen I. Institutional Special Needs Plans: 2024 Market Landscape and Future Considerations. https://www.milliman.com/en/insight/institutional-special-needs-plans-2024-market-landscape-future.

[8] Singletary E., Roiland R., Harker M., Taylor D., Saunders R. (2021). Value Based Payment and Skilled Nursing Facilities: Supporting SNFs During COVID-19 and Beyond.

[9] Page M.J., McKenzie J.E., Bossuyt P.M., Boutron I., Hoffmann T.C., Mulrow C.D., Shamseer L., Tetzlaff J.M., Moher D. (2021). Updating Guidance for Reporting Systematic Reviews: Development of the PRISMA 2020 Statement. J. Clin. Epidemiol..

[10] Kruse C.S. (2019). Writing a Systematic Review for Publication in a Health-Related Degree Program. JMIR Res. Protoc..

[11] Arksey H., O’Malley L. (2005). Scoping Studies: Towards a Methodological Framework. Int. J. Soc. Res. Methodol..

[12] Dhingra L., Lipson K., Dieckmann N.F., Chen J., Bookbinder M., Portenoy R. (2019). Institutional Special Needs Plans and Hospice Enrollment in Nursing Homes: A National Analysis. J. Am. Geriatr. Soc..

[13] Signature Advantage Signature Advantage Advantage Plan & Signature Advantage Community (HMO ISNP) Provider Manual 2021. https://signatureadvantageplan.com/wp-content/uploads/documents/2021/2021%20Provider%20Manual%20Final.pdf.

[14] Chen A.C., Hnath J.G.P., Grabowski D.C. (2024). Institutional Special Needs Plans In Nursing Homes: Substantial Enrollment Growth But Low Availability, 2006–21. Health Aff..

[15] White L.L.Y., Sun C., Coe N.B. (2024). Quality of Hospices Used by Medicare Advantage and Traditional Fee-for-Service Beneficiaries. JAMA Netw. Open.

[16] Chen A.C., Grabowski D.C. (2025). A Model to Increase Care Delivery in Nursing Homes: The Role of Institutional Special Needs Plans. Health Serv. Res..

[17] Yun H., Rahman M., McGarry B., White E.M., Meyers D.J., Kosar C.M. (2025). Disenrollment from Special Needs and Other Medicare Advantage Plans Among Nursing Home Residents. JAMA Netw. Open.

[18] Rahman M., McGarry B., White E.M., Grabowski D.C., Kosar C.M. (2025). Is Managed Care Effective in Long-Term Care Settings? Evidence from Medicare Institutional Special Needs Plans.

